# Use of Web-Based Surveys to Collect Long-Term Pediatric Outcomes in Patients With Twin-Twin Transfusion Syndrome Treated With Fetoscopic Laser Photocoagulation: Observational Study

**DOI:** 10.2196/60039

**Published:** 2024-09-11

**Authors:** Eric Bergh, Kimberly Rennie, Jimmy Espinoza, Anthony Johnson, Ramesha Papanna

**Affiliations:** 1 Division of Fetal Intervention, Department of Obstetrics, Gynecology and Reproductive Sciences, McGovern Medical School at UTHealth Houston, 6410 Fannin Street, Suite 201, Houston, TX, 77030, United States, 1 (832) 325 7288, 1 713 383 1464; 2 College of Letters and Science, Psychology, University of Wisconsin Milwaukee, Milwaukee, WI, United States

**Keywords:** automation, REDCap, data collection, reporting, response rate, response rates, survey, surveys, questionnaire, questionnaires, fetal medicine, pediatric outcomes, long-term outcomes, photocoagulation, twin, twins, blood, pregnant, pregnancy, pediatric, pediatrics, infant, infants, infancy, baby, babies, neonate, neonates, neonatal, newborn, newborns, maternal, in utero, TTTS, fetus, fetal, twin-twin transfusion syndrome

## Abstract

**Background:**

In the United States, patients with monochorionic diamniotic twins who undergo in utero fetoscopic laser photocoagulation (FLP) for twin-twin transfusion syndrome (TTTS) may travel great distances for care. After delivery, many parents cannot return to study sites for formal pediatric evaluation due to geographic location and cost.

**Objective:**

The aim of this study was to collect long-term pediatric outcomes in patients who underwent FLP for TTTS.

**Methods:**

We assessed the feasibility of using a web-based survey designed in REDCap (Research Electronic Data Capture; Vanderbilt University) to collect parent-reported outcomes in children treated for TTTS at a single center during 2011‐2019. Patients with ≥1 neonatal survivor were invited via email to complete 5 possible questionnaires: the child status questionnaire (CSQ); fetal center questionnaire (FCQ); Ages & Stages Questionnaires, Third Edition (ASQ-3); Modified Checklist for Autism in Toddlers, Revised With Follow-Up (M-CHAT-R/F); and thank you questionnaire (TYQ). The R programming language (R Foundation for Statistical Computing) was used to automate survey distribution, scoring, and creation of customized reports. The survey was performed in 2019 and repeated after 12 months in the same study population in 2020.

**Results:**

A total of 389 patients in 26 different states and 2 international locations had an email address on file and received an invitation in 2019 to complete the survey (median pediatric age 48.9, IQR 1.0‐93.6 months). Among surveyed mothers in 2019, the overall response rate was 37.3% (145/389), and the questionnaire completion rate was 98% (145/148), 87.8% (130/148), 71.1% (81/100), 86.4% (19/22), and 74.3% (110/148) for the CSQ, FCQ, ASQ-3, M-CHAT-R/F, and TYQ, respectively. In 2020, the overall response rate was 57.8% (56/97), and the questionnaire completion rate was 96.4% (54/56), 91.1% (51/56), 86.1% (31/36), 91.7% (11/12), and 80.4% (45/56) for the CSQ, FCQ, ASQ-3, M-CHAT-R/F, and TYQ, respectively.

**Conclusions:**

This is the first study to use both REDCap and computer automation to aid in the dissemination, collection, and reporting of surveys to collect long-term pediatric outcomes in the field of fetal medicine.

## Introduction

### Background

Twin-twin transfusion syndrome (TTTS) results from unbalanced vascular communications in a shared placenta between monochorionic diamniotic (MCDA) twins. Bidirectional vascular communications are present in up to 95% of MCDA twins and allow for a single, shared circulatory system [1]. However, in 9%‐15% of MCDA twins, unbalanced vascular communications produce a pathological state in which one fetus (the donor twin) overtransfuses the cotwin (the recipient) [2,3]. Compensatory mechanisms result in progressive recipient polyhydramnios (excess amniotic fluid) and donor oligohydramnios (low amniotic fluid). Expectant management of this condition results in a mortality rate greater than 70%, typically due to sequelae from circulatory overload, compensatory hormonal dysfunction, and preterm delivery due to worsening polyhydramnios [4]. The gold-standard treatment for TTTS is intrauterine fetoscopic laser photocoagulation (FLP) of placental vascular anastomoses, which halts the abnormal blood exchange and yields better outcomes [5]. Despite this therapy, twins who survive TTTS may experience major disability at birth likely secondary to hemodynamic changes that occur in utero or sequelae of prematurity, as the average gestational age at delivery is approximately 32 weeks [6]. However, long-term outcomes for these surviving twins in the United States remain understudied, largely because of the logistical challenges of following patients who traveled far from home for treatment.

### Prior Work

The data on long-term pediatric outcomes in patients who undergo FLP for TTTS come almost entirely from centers outside the United States. Centers with a local and homogeneous referral base are more likely to report in-person pediatric evaluations [7-10], although telephone and mail-in surveys have also been reported [9,11,12]. To date, there have been few attempts to collect long-term pediatric outcomes using web-based methods in the field of fetal surgery [13].

In the United States, patients referred for treatment of TTTS may travel upward of 2000 miles to receive care at a tertiary center of excellence [14]. The majority of these patients will travel home postprocedure and deliver at remote sites, which makes tracking neonatal and long-term outcomes challenging. At our center, thanks to considerable efforts from full-time research staff to collect maternal delivery and neonatal discharge records from patients’ delivering hospitals, we have reported on the immediate and short-term neonatal complications (from time of delivery until hospital discharge) in patients who undergo FLP for TTTS [15]. However, prospectively collected in-person long-term follow-up of twins born after FLP in the United States would be exceedingly challenging and resource-intensive. Therefore, web-based collection methods may provide a viable approach.

### Goal of This Study

The primary outcome of this study was to assess the feasibility of using computer automation to obtain, to the fullest extent, long-term pediatric outcomes from patients who underwent FLP for TTTS at a fetal center (FC) over a 2-year period.

## Methods

### Ethical Considerations

Approval was obtained from the Institutional Human Research Ethics Committee (IRB) (HSC-MS-19‐0363), and the study was conducted between June 1, 2019, and September 30, 2020. The IRB determined that our study did not need ethical approval.

### Study Design

This was a cohort study of patients who were referred to the UTHealth Houston Fetal Center in Houston, Texas, and who underwent FLP for TTTS between 2011 and 2019. Eligible patients were identified retrospectively from a registry of patients treated at our center who had previously consented to prospective follow-up of short-term maternal and neonatal outcomes (HSC-MS-10‐0059).

Patients with TTTS who underwent FLP at our center during the study period and had both an email address on file and at least 1 surviving child from a monochorionic pair at the time of neonatal hospital discharge were eligible for participation in this study. Exclusion criteria included patients without a registered email or cases of dual fetal or neonatal demise.

### Patient Recruitment and Consent

Patients who received the survey via email were instructed to follow a hyperlink to a web-based REDCap (Research Electronic Data Capture; Vanderbilt University) consent form, where details regarding study participation and confidentiality were provided. After giving e-consent, patients were emailed copies of the study protocol and directed to a subsequent child status questionnaire (CSQ). After indicating the survival status of both the ex-donor and the ex-recipient twins, the user was directed to a queue of web-based questionnaires, specific to the number of surviving children and their age.

### Questionnaires

We designed research surveys in REDCap, an HIPAA (Health Insurance Portability and Accountability Act)–compliant, secure research data collection tool, which can be used to distribute web-based, mobile-friendly surveys. Surveys consisted of several pediatric age–specific questionnaires distributed via email to consenting parents. Details regarding the purpose, age applicability, and atypical screening threshold are present in Table 1.

**Table 1.** Summary of questionnaires in surveys distributed to participants.^a^

Questionnaire	Purpose	Applicability	Atypical screening threshold
CSQ^b^^,^^c^	Confirm child survival status Confirm if the child was the ex-recipient or ex-donor twin	Single questionnaire delivered to all consenting patients	N/A^d^
FCQ^e^	20 questions with “yes/no” responses related to general health and the use of special services	Parent-reported questionnaire for each child regardless of current pediatric age	N/A
ASQ-3^f^	40 questions with “yes/no” or “yes/sometimes/not yet” responses completed by parents designed to detect developmental delays in 5 domains: communication, gross-motor, fine-motor, problem-solving, and personal-social	Parent-reported questionnaire for each child between the ages of 1 and 60 months	Score of greater than or equal to 2 SD below the mean on any of the 5 domains assessed
M-CHAT-R/F^g^	20 questions with “yes/no” responses completed by parents to screen for autism spectrum disorder	Parent-reported questionnaire for each child between the ages of 16 and 30 months	Atypical response in 3 or more questions
TYQ^h^	Obtain permission to distribute repeat survey in 12 months Obtain appropriate contact information	Single questionnaire delivered to all consenting participants	N/A

a All questionnaires delivered via email through REDCap (Research Electronic Data Capture). Does not include the consent questionnaire, which was distributed to all eligible patients via email through REDCap and used the REDCap e-consent framework.

b CSQ: child status questionnaire.

c Age corrected for prematurity until 24 months of age.

d N/A: not applicable.

e FCQ: fetal center questionnaire.

f ASQ-3: Ages & Stages Questionnaires, Third Edition

g M-CHAT-R/F: Modified Checklist for Autism in Toddlers, Revised With Follow-Up.

h TYQ: Thank You Questionnaire.

First, all participating patients were sent the CSQ, a series of 2‐4 questions for each child, which provided confirmation of the child status as alive, demised in utero (fetal demise), or demised after birth (neonatal demise). The parent also reported the recipient or donor status for each child so that prenatal parameters could be accurately correlated with the correct twin. Based on user input indicating both the number of surviving children and pediatric age, specifically designed computer algorithms automatically tailored the number and type of survey questionnaires. Participating parents were sent any questionnaires applicable to their child’s pediatric age.

The fetal center questionnaire (FCQ) was adapted from a prior publication of long-term outcomes in twin gestations and was applicable to every surviving child [16]. The questionnaire consisted of 20 questions, the majority with “yes/no” responses, related to general health and the use of specialized services related to movement, speech, hearing, behavior, and education. The complete questionnaire is included in Multimedia Appendix 1.

The Ages & Stages Questionnaires, Third Edition (ASQ-3), a validated developmental screening tool with approximately 40 “yes/no” or “yes/sometimes/not yet” questions designed to be completed by parents, was applicable to every child between the ages of 1 and 60 months. This evaluation tool has high sensitivity and specificity to detect developmental delays in 5 domains: communication, gross-motor, fine-motor, problem-solving, and personal-social [17-21]. After obtaining permission from the publishers, we integrated all 21 age-specific versions of the ASQ-3 into REDCap as separate questionnaires.

The Modified Checklist for Autism in Toddlers, Revised With Follow-Up (M-CHAT-R/F), a validated autism screening tool with 20 “yes/no” questions designed to be completed by parents, was applicable to children between the ages of 16 and 30 months [22,23]. With permission from the publishers, the M-CHAT-R/F was distributed as a REDCap questionnaire. Participants whose children had a positive M-CHAT-R/F screen received a phone call and completed a series of follow-up questions per screening protocol.

The ASQ-3 and M-CHAT-R/F have been tested and validated in populations of children who are at risk for prematurity, autism, and abnormal neurodevelopmental outcomes [17-23]. They were selected for this survey due to ease of completion and ease of distribution as REDCap questionnaires.

Finally, all surveys were finished with a brief thank you questionnaire (TYQ). The TYQ consisted of 5 free text and “yes/no”-style questions requesting the following: permission to send a repeat survey 1 year later, the patient’s preferred method of contact for future studies, and permission to contact the patient to validate any results or obtain prior results from their child’s pediatrician’s office.

### Distribution

The current age of all children surveyed was calculated, adjusting for prematurity until 24 months. For children who were older than 60 months, the CSQ, FCQ, and TYQ were distributed at a single timepoint. For children who were eligible for the ASQ-3, the survey was automatically distributed on a rolling basis approximately 3 weeks prior to the date at which a child’s age-specific ASQ-3 would no longer be applicable. Depending upon the child’s age and the number of surviving children (determined via the CSQ), parents’ survey queue (not including the single CSQ and TYQ) could contain as few as 1 FCQ questionnaire (eg, 1 surviving child at 6 years of age) and as many as 6 questionnaires (eg, 2 living children at 24 months of age). Patients who did not respond to the initial survey invitation within 1 week received 2 additional weekly reminders via email, followed by a phone call.

### Scoring and Questionnaire Reporting

Scripts in the R programming language (R Foundation for Statistical Computing) were used to automatically score completed ASQ-3, accounting for an age-specific scoring rubric and adjustment for any skipped questions, and the M-CHAT-R/F. The ASQ-3 was considered high risk if any of the 5 domains assessed scored greater than or equal to 2 SDs below the mean. The M-CHAT-R/F examination was considered high risk if 3 or more questions had an atypical response. R language scripting was also used to both identify any child who had a high-risk ASQ-3 or M-CHAT-R/F screen and to automate the creation of custom reports in Microsoft Word (Microsoft Corp) using the *WordR* [24] and *officer* [25] packages. These reports were distributed to parents via HIPAA secure email within 2 weeks of survey completion.

### Repeat Surveys

Patients who indicated they were amenable to a repeat survey received a second survey invitation in 2020 approximately 12‐13 months after their 2019 response. All applicable age-specific questionnaires were repeated as part of a prospective analysis of pediatric developmental outcomes in this population. As part of the repeat 2020 survey, patients received a single email reminder but did not receive phone calls due to a lack of available research staffing. Questionnaires and follow-up reports were generated and distributed automatedly within 2 weeks of survey completion.

### Statistical Analysis: Power

As a purely observational study, no official power analysis was performed, as the primary objective was to collect, to the fullest extent, parent-reported long-term outcomes in this population over a 2-year period.

## Results

**Figure 1.**
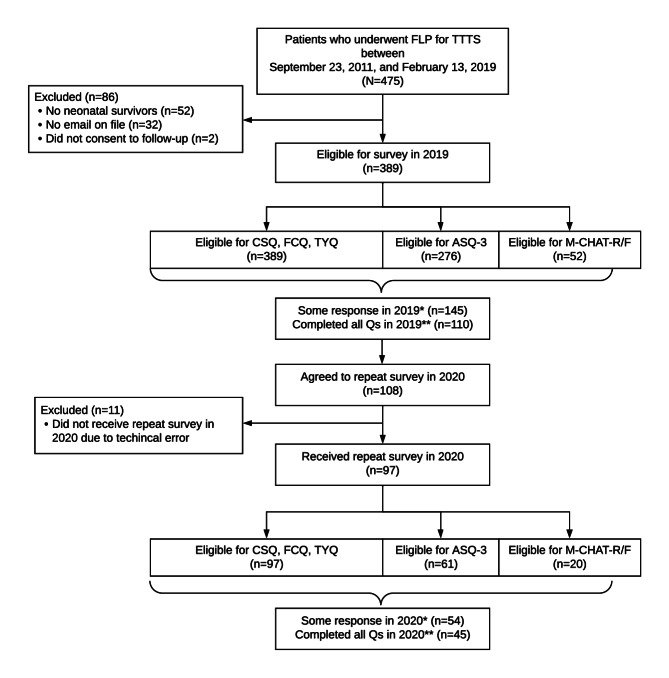
Patient recruitment and responses. ASQ-3: Ages & Stages Questionnaires, Third Edition; CSQ: child status questionnaire; FCQ: fetal center questionnaire; FLP: fetoscopic laser photocoagulation; M-CHAT-R/F: Modified Checklist for Autism in Toddlers, Revised With Follow-Up; TTTS: twin-twin transfusion syndrome; TYQ: thank you questionnaire. *Some response to any of CSQ, FCQ, ASQ-3, or M-CHAT-R/F. **Completed all age-appropriate questionnaires (Qs).

Figure 1 is a flow diagram of patient recruitment and responses. In total, 475 patients underwent FLP for TTTS at our center between September 23, 2011, and February 13, 2019. Among them, 86 patients were excluded from the study: 52 had no neonatal survivors, 32 did not have an email on file at the time of the initial survey, and 2 did not consent to be contacted for future studies. A total of 389 mothers met the inclusion criteria and received an invitation via email to participate in the 2019 survey. Of these, 148 (38%) patients signed consent, and 145 patients completed or partially completed the full survey (CSQ, FCQ, ASQ-3, M-CHAT-R/F, or TYQ; 37.3% response rate). A total of 108 patients who responded in 2019 agreed to a repeat survey in 2020, of which 11 participants did not receive a repeat survey due to a technical error. In 2020, 97 patients from the 2019 cohort received a repeat survey, of whom 56 patients signed consent (57.8%) and 54 patients completed or partially completed the full set of questionnaires (55.7% response rate).

The majority of patients surveyed in 2019 and 2020 were from the southern United States (2019: 336/389, 86.8% and 2020: 80/97, 82.5%), had 2 neonatal survivors (2019: 325/389, 83.5% and 2020: 82/97, 84.5%), and underwent FLP for stage III TTTS (2019: 205/389, 52.4% and 2020: 52/97, 52.6%; Table 2). Slightly more than half of patients each year were from Texas (2019: 215/389, 55.3% and 2020: 51/97, 52.6%), and relatively few delivered within the same hospital system as the FC (2019: 41/389, 10.5% and 2020: 8/97, 8.2%). Compared with nonresponders, patients who consented to participate in the 2019 survey were older (30.0 vs 28.0 years; *P*<.001) and had younger children at the time of the survey (42.0 vs 52.0 months of age; *P*=.005), were less likely to have male children (68/145, 46.9% vs 143/244, 58.6%; *P*=.02), had a higher incidence of coexisting twin-anemia polycythemia sequence (10/141, 7.1% vs 5/239, 2.1%; *P*=.02), and had lower rates of coexisting selective fetal growth restriction (51/144, 35.2% vs 114/244, 46.7%; *P*=.03) and donor twins weighed more at delivery (1360 g vs 1570 g; *P*=.03). In both 2019 and 2020, there were no differences between responders and nonresponders with respect to race, number of surveys to complete, incidence of triplet pregnancies, TTTS Quintero stage, cervical length, gestational age at FLP, gestational age at delivery, child survival status, region of origin, distance from the FC, or incidence of delivery within the FC hospital system.

The completion rates and time to completion based on recorded survey timestamps in REDCap for each questionnaire are listed in Table 3. Reported completion times are limited to less than 60 minutes to exclude outliers who opened the questionnaire and completed it later. In 2019, 38% of eligible patients signed consent. In total, 98% (145/148) of consenting and eligible participants completed the CSQ, 87.8% (130/148) completed the FCQ in a median (IQR) of 3 (2-5) minutes, 71.1% (81/100) completed the ASQ-3 in 4 (3-8) minutes, and 86.4% (19/22) completed the M-CHAT-R/F in 2 (1-3) minutes. Of the patients who signed consent, 74.3% (110/148) completed the entire survey. Among patients who received a repeat survey in 2020, 57.8% (56/97) of patients signed consent. In total, 96.4% (54/56) of consenting and eligible participants completed the CSQ, 91.1% (51/56) completed the FCQ in a median (IQR) of 3 (2-5) minutes, 86.1% (31/36) completed the ASQ-3 in 6 (4‐8.5) minutes, and 91.7% (11/12) completed the M-CHAT-R/F in 2 (1-3) minutes. Of the patients who signed consent, 80.4% (45/56) completed the entire survey.

The rates of atypical developmental screens are listed in Table 4. When analysis of the ASQ-3 was limited to the oldest assessment performed at ≥24 months of age for each child obtained in either 2019 or 2020, the overall rate of atypical ASQ-3 was 18.9% (18/95; recipient twin: 9/46, 19.6% and donor twin: 9/49, 18.4%). In 2019 and 2020, the rate of atypical M-CHAT-R/F screens was 11.1% (2/18) and 18.2% (2/11), respectively, for ex-recipient twins and 6.67% (1/15) and 9.1% (1/11), respectively, for ex-donor twins. In 2019 and 2020, the follow-up M-CHAT-R/F telephone confirmation was not performed for 3 children (total 2 recipient and 1 donor) due to the inability to reach the patient.

Patient willingness to undergo repeat survey and their preferred method of communication are listed in Table 5. Nearly 100% of patients in both years were amenable to a repeat survey in the following year. Respectively, most patients in 2019 and 2020 indicated a preference for email communication (99/110, 90% vs 43/45, 95.6%), followed by telephone calls (44/110, 40% vs 17/45, 37.8%), and finally mail-in post (24/110, 21.8% vs 11/45, 24.4%). In 2019 and 2020, 92.7% (102/110) and 95.6% (43/45) of responders were amenable to a follow-up call to sign a release of information waivers to request medical records from their child’s pediatrician’s office.

**Table 2.** Baseline characteristics of survey participants stratified by response.

Characteristic		2019			2020^a^		
		No response(n=244)	Some response (n=145)	*P* value^b^	No response(n=43)	Some response (n=54)	*P* value^b^
Maternal age (years), median (IQR)		28.0 (23.0-31.0)	30.0 (27.0-35.0)	*<.001^c^*	30.0 (27.0-33.5)	30.5 (27.0-34.0)	.50
Pediatric age (months, corrected)^d^, median (IQR)		52 (32-72)	42 (17-64)	*.005*	55 (33-86)	51 (32-77)	.40
**Race, n (%)**				.13			.31
	African American	29 (11.9)	8 (5.5)		2 (4.7)	2 (3.7)	
	Asian	6 (2.5)	4 (2.8)		1 (2.3)	0 (0)	
	Hispanic	57 (23.4)	27 (18.6)		10 (23.3)	7 (13)	
	White	150 (61.5)	105 (72.4)		30 (69.8)	45 (83.3)	
	Other	2 (0.8)	1 (0.7)		0 (0)	0 (0)	
**Questionnaire count** ^e^ **, n (%)**				.11			.55
	3	12 (4.9)	5 (3.4)		3 (7)	3 (5.6)	
	4	92 (37.7)	37 (25.5)		17 (39.5)	20 (37)	
	5	9 (3.7)	5 (3.4)		2 (4.7)	0 (0)	
	6	110 (45.1)	81 (55.9)		15 (34.9)	19 (35.2)	
	8	21 (8.6)	17 (11.7)		6 (14)	12 (22.2)	
Sex: male, n (%)		143 (58.6)	68 (46.9)	*.02*	19 (44.2)	30 (55.6)	.27
**Twin type, n (%)**				.22			.63
	MCDA^f^	234 (95.9)	138 (95.2)		42 (97.7)	50 (92.6)	
	MCMA^g^	0 (0)	2 (1.4)		0 (0)	2 (3.7)	
	Higher order	10 (4.1)^h^	5 (3.4)		1 (2.3)	2 (3.7)	
Triplets, n (%)		9 (3.7)	5 (3.4)	>.90	1 (2.3)	2 (3.7)	>.99
**Triplet type, n (%)**				>.99			.33
	DCTA^i^	8 (88.9)	4 (80)		0 (0)	2 (100)	
	MCTA^j^	1 (11.1)	1 (20)		1 (100)	0 (0)	
**TTTS^k^ Quintero stage, n (%)**				.74			>.90
	I	33 (13.5)	17 (11.7)		3 (7)	7 (13)	
	II	64 (26.2)	45 (31)		14 (32.6)	16 (29.6)	
	III	133 (54.5)	72 (49.7)		24 (55.8)	28 (51.9)	
	IV	9 (3.7)	7 (4.8)		2 (4.7)	2 (3.7)	
	Isolated TAPS^l^^,^^m^	5 (2)	4 (2.8)		0 (0)	1 (1.9)	
TTTS+TAPS^m^, n (%)		5 (2.1)	10 (7.1)	*.02*	3 (7)	2 (3.8)	0.65
TTTS+sFGR^n^^,^^o^, n (%)		114 (46.7)	51 (35.2)	*.03*	18 (41.9)	20 (37)	.63
**FI**^p^ **procedure, n (%)**				.29			.63
	FLP^q^ (percutaneous)	226 (92.6)	138 (95.2)		42 (97.7)	50 (92.6)	
	FLP (laparoscopic-assisted)	16 (6.6)	5 (3.4)		1 (2.3)	2 (3.7)	
	Selective reduction	1 (0.4)^r^	2 (1.4)^s^		0 (0)	2 (3.7)^s^	
	Failed laser, amnioreduction only	1 (0.4)^t^	0 (0)		0 (0)	0 (0)	
Required repeat procedure, n (%)		2 (0.8)^u^	3 (2.1)^v^	.37	2 (4.7)^w^	1 (1.9)^x^	.58
Intertwin weight discordance (%)^y^, median (IQR)		25 (16-34)	22 (13-30)	*.02*	21 (16-32)	22 (14-31)	.76
Cervical length (mm), median (IQR)		38 (31-45)	40 (32-47)	.26	42 (33-47)	39 (29-48)	.41
GA^z^ at FLP (weeks), median (IQR)		20.43 (18.82-22.29)	20.43 (18.71-22.29)	.82	20.29 (18.36-22.14)	20.14 (18.75-22.25)	>.99
GA at delivery (weeks), median (IQR)		32.0 (29.4-34.1)	32.3 (29.4-34.3)	.49	32.86 (31.00-34.57)	32.00 (29.46-34.68)	.22
Recipient birth weight (g), median (IQR)		1725 (1283-2139)	1778 (1391-2193)	.22	1860 (1625-2248)	1830 (1326-2171)	.40
Donor birth weight (g), median (IQR)		1360 (975-1890)	1570 (1170-2000)	*.03*	1730 (1300-2094)	1570 (1170-2020)	.57
**Child status, n (%)**				.17			.21
	Ex-donor loss	40 (16.4)	14 (9.7)		7 (16.3)	5 (9.3)	
	Dual survivors	198 (81.1)	127 (87.6)		36 (83.7)	46 (85.2)	
	Ex-recipient loss	6 (2.5)	4 (2.8)		0 (0)	3 (5.6)	
**Region of origin, n (%)**				.12			.44
	Midwest	21 (8.7)	9 (6.2)		2 (4.7)	5 (9.3)	
	South	212 (87.6)	124 (85.5)		38 (88.4)	42 (77.8)	
	West	9 (3.7)	12 (8.3)		3 (7)	7 (13)	
Texas based, n (%)		134 (54.9)	81 (55.9)	.86	24 (55.8)	27 (50)	.57
Distance from FC^aa^ (miles), median (IQR)		266 (189-454)	237 (150-489)	.12	237 (151-590)	232 (169-637)	.68
Delivered within FC hospital system, n (%)		24 (9.8)	17 (11.7)	.56	5 (11.6)	3 (5.6)	.46

a Represents a subgroup of patients initially surveyed in 2019 who indicated they were amenable to repeat survey in 2020.

b Wilcoxon rank sum test, Fisher exact test, and Pearson chi-square test.

c Values in italics format indicate *P*<.05.

d Pediatric age corrected for prematurity until 24 months of age.

e Number of questionnaires to be performed based on child’s age at the time of survey. Does not include child status questionnaire and thank you questionnaire.

f MCDA: monochorionic diamniotic.

g MCMA: monochorionic monoamniotic.

h All triplet gestations with the exception of a single quadruplet (dichorionic quadramniotic, nonrespondent).

i DCTA: dichorionic triamniotic.

j MCTA: monochorionic triamniotic.

k TTTS: twin-twin transfusion syndrome.

l TAPS: twin anemia polycythemia sequence.

m Defined as delta middle cerebral artery peak systolic value (MCA-PSV) ≥1. Excludes cases of isolated TAPS.

n sFGR: selective fetal growth restriction.

o Defined as ≥25% intertwin estimated weigh discordance and either donor or recipient twin estimated fetal weight <10 percentile at FI.

p FI: fetal intervention.

q FLP: fetoscopic laser photocoagulation.

r MCDA pregnancy in which cords too close for FLP, bipolar cord coagulation performed.

s Monochorionic monoamniotic with TTTS converted to bipolar after FLP (n=1); TTTS with sFGR, primary radiofrequency ablation of donor twin (n=1).

t Quintero stage II with poor visualization on diagnostic fetoscopy secondary to prior bleed, amnioreduction only performed (n=1).

u Quintero stage II FLP at 18 wk and 0 d, followed by recurrent TTTS or TAPS and ventriculomegaly in donor at 19 wk and 6 d and underwent radiofrequency ablation (n=1); Quintero stage II FLP for DCTA triplet gestation at 18 wk and 3 d with radiofrequency ablation of donor due to recurrent TTTS or TAPS at 20 wk and 1 d (n=1).

v Quintero stage III TTTS FLP at 21 wk and 2 d with recurrent TTTS at 24 wk and 5 d and underwent repeat FLP (n=1); Quintero stage IV at 16 wk and 3 d with recurrent TTTS at 24 wk and 0 d underwent repeat FLP (n=1); Quintero stage II TTTS FLP at 18 wk and 2 d with recurrent TAPS and amniotic band at 20 wk and 0 d had repeat FLP and amniotic band lysis from neck of plethoric fetus (n=1).

w Quintero stage III TTTS at 21 wk and 2 d with recurrent stage III TTTS underwent repeat FLP at 24 wk and 5 d (n=1); Quintero stage II TTTS FLP at 18 wk and 2 d with recurrent TAPS and amniotic band at 20 wk and 0 d had repeat FLP and amniotic band lysis from neck of plethoric fetus (n=1).

x Quintero stage IV FLP at 16 wk and 3 d with recurrent TTTS and repeat FLP at 24 wk and 0 d.

y Defined as intertwin estimated fetal weight discordance of ≥25% and either the donor or recipient estimated fetal weight <10%.

z GA: gestational age.

aa FC: fetal center.

**Table 3.** Survey response rate^a^ among patients who had fetoscopic laser photocoagulation between September 23, 2011, and February 13, 2019.

	Eligible	Completed (% response rate)	Completion time (minutes)^b^, median (IQR)
**Questionnaire (2019 survey)**			
Consent	389	148 (38)	—^c^
CSQ^d^	148^e^	145 (98)	—
FCQ^f^^,g^	148^e^	130^h^ (87.8)	3 (2-5)
ASQ-3^i^^,j^	100^e^	81^h^(71.1)	5 (3-8)
M-CHAT-R/F^h^^,k^	22^e^	19 (86.4)	2 (1-3)
TYQ	148^e^	110 (74.3)^l^	—
**Questionnaire (2020 survey)** ^m^			
Consent	97	56 (57.8)	—
CSQ	56^e^	54 (96.4)	—
FCQ^g^	56^e^	51^n^ (91.1)	3 (2-5)
ASQ-3^j^	36^e^	31 (86.1)	6 (4-8.5)
M-CHAT-R/F^h^	12^e^	11 (91.7)	2 (1-3)
TYQ	56^e^	45 (80.4)^l^	—

a Counts represent number of patients surveyed.

b Excluding outliers >60 minutes; time to complete the consent, CSQ, and TYQ was not recorded.

c Not available.

d CSQ: child status questionnaire.

e Represents the number of patients who signed consent and eligible for the survey.

f FCQ: fetal center questionnaire.

g All mothers eligible.

h Does not include 1 partial response.

i ASQ-3: Ages & Stages Questionnaires, Third Edition.

j Patients with children between ages 1 and 60 months eligible.

k M-CHAT-R/F: Modified Checklist for Autism in Toddlers, Revised With Follow-Up.

l Represents the percentage of patients who signed consent who finished the entire survey.

m Represents a subgroup of patients initially surveyed in 2019 who indicated they were amenable to repeat survey in 2020.

n Does not include 2 partial response.

**Table 4.** Atypical developmental screens in 2019 and 2020 by twin.

	Twin		Total, n (%)
Ex-recipient, n (%)	Ex-donor, n (%)
**ASQ** **-3^a^ (2019+2020 survey)≥24 months of age^b^**			
Typical	37 (80.4)	40 (81.6)	77 (80.2)
Atypical^c^	9 (19.6)	9 (18.4)	18 (18.9)
**M-CHAT-R/F^d^ questionnaire (2019 survey)**			
Typical	16 (88.9)	14 (93.3)	30 (90.9)
Atypical^e^	2 (11.1)^e^	1 (6.67)	3 (9.1)
**M-CHAT-R/F questionnaire (2020 survey)**			
Typical	9 (81.8)	10 (90.9)	19 (86.4)
Atypical^f^	2 (18.2)^f^	1 (9.1)^g^	3 (13.6)

a ASQ-3: Ages & Stages Questionnaires, Third Edition.

b Represents the oldest ASQ-3 assessment performed at ≥24 months of age for each child obtained in either 2019 or 2020.

c Indicates at least 1 domain on the ASQ-3 for which the score was ≥2 SD below the mean.

d M-CHAT-R/F: Modified Checklist for Autism in Toddlers, Revised With Follow-Up.

e Indicates 3 or more atypical responses.

f Unable to contact a single patient to confirm atypical response.

g Unable to contact a single patient (flagged recipient and donor) to confirm atypical response.

**Table 5.** Results of thank you questionnaire.

Survey year	Ok to contact for repeat survey?, n (%)	Preferred method of future contact			Ok to inquire about pediatrician records?, n (%)
		Telephone, n (%)	Email, n (%)	Post^a^, n (%)	
2019	109 (99.1)	44 (40.4)	99 (90.8)	24 (21.8)	102 (92.7)
2020^b^	45 (100)	17 (37.8)	43 (95.6)	11 (24.4)	43 (95.6)

a Two patients who requested communication via post and provided their current home address had moved 115 and 1577 miles since the time of initial evaluation for FLP.

b Represents a subgroup of patients initially surveyed in 2019 who indicated they were amenable to repeat survey in 2020.

## Discussion

### Principal Findings

In this survey study, we effectively gathered long-term pediatric parent-reported outcomes in patients treated with FLP for TTTS via email and electronic questionnaires. The overall response rates to our survey were 37.3% (145/389) in 2019 and 55.7% (56/97) in 2020. Notably, slightly less than half of the patients who responded were from outside of Texas (in 2019: 64/145, 44.1% and in 2020: 27/54, 50%), and the majority (in 2019: 128/145, 88.3% and in 2020: 51/54, 94.4%) delivered outside the FC hospital system. Of the patients who consented to our survey, the overall completion rate was 74.3% (110/148) in 2019 and 80.4% (45/56) in 2020.

### Comparison With Prior Work

Compared with patients in Europe, the long-term pediatric outcomes in patients who travel for the treatment of TTTS in the United States have been poorly studied. Several Western European centers in which centralized health care systems exist have reported on the in-person evaluation of large cohorts of pediatric survivors of TTTS with nearly 100% follow-up rates [8,26,27]. Conversely, there is but a single report of long-term cognitive outcomes in children treated for TTTS and assessed solely in the United States [7]. In that study, only 13% of patients from outside the study center state were available for in-person assessment. This lack of data on long-term outcomes represents a critically missing component with which to counsel patients who are evaluated and treated for TTTS in the United States.

There are several challenges that contribute to the difficulty in assessing long-term pediatric outcomes in patients treated for TTTS in the United States. First, the geographic distance patients travel for specialized fetal intervention care is a physical barrier to in-person follow-up [14]. As a niche specialty, few high-volume academic centers account for the majority of FLP procedures performed annually [28]. Consequently, patients who reside outside these locations will travel great distances to receive care during their pregnancy, only to return home for follow-up care. This is evidenced by the geographic distance from our center in the population of patients surveyed and the high proportion of patients who delivered outside the FC hospital system.

Second, compared with other high-income countries, the United States ranks last regarding measures of health care affordability and access to care [29]. As of 2023, approximately 25.3 million people were uninsured in the United States [30]. Furthermore, as of 2016, there were 626 individual health systems identified across the United States [31]. Both the lack of access to care and the complex system of health care networks may contribute to the challenges in the longitudinal assessment of pediatric patients.

Finally, in most health systems in the United States, a fetus is not assigned a medical record number despite being exposed to disease, medications, and even fetal surgical interventions prior to birth. Both technical and legal challenges have likely contributed to the barriers surrounding the creation of a fetal electronic health record. Historically, fetal data are linked to pediatric outcomes via the maternal chart, so any attempt to fully describe an individual’s medical history, from the time of conception to pediatric and adult life, requires the additional step of linking these 2 individuals. Despite some recent strategies to create nested or embedded fetal records within a maternal record [32], this has not been universally adopted in the field of obstetrics and fetal surgery.

Considering the challenges in obtaining long-term pediatric outcomes in patients treated for TTTS, validated parent-reported screening questionnaires delivered via electronic media are a potential starting point toward addressing this problem. We acknowledge that the gold standard for pediatric neurodevelopmental evaluation is in-person assessment. However, in the context of a population of individuals spread across a large geographic area and among various health care networks with diverse levels of access to care, remote screening provides a unique opportunity to assess outcomes in this population.

The use of electronic patient-reported outcome tools has increased with the growth of electronic health technologies. Significant advantages of this strategy include the ability to obtain information remotely over great distances and doing so at relatively low cost with the help of computer programming and automation. Furthermore, the real-time analysis of patient-reported data allows for early detection of positive screens and improvements in patient-clinician communication [33]. In our study, we developed scripts to automatically generate both accurate and personalized reports for children who had positive screens, which were subsequently returned to participants to share with their primary physician, thereby illustrating the potential clinical utility of this tool.

### Strengths and Limitations

To our knowledge, this is the first report in fetal medicine of the ASQ-3 and M-CHAT-R/F being delivered to study participants via the REDCap questionnaire. Previous studies of survivors of TTTS describe the distribution of the ASQ-3 to patients via post [34-37], email attachment [34], or telephone interview [34], but incorporating these surveys into a digital format for research in fetal medicine has not been previously reported. This novel approach allowed for the application of computer-based algorithms to schedule the timely distribution of surveys, automate questionnaire scoring, and generate individualized reports for atypical screening responses. Furthermore, the costs to implement this system within an academic university hospital system where REDCap is an established research tool were minimal and involved licensing of the ASQ-3 for distribution as a research questionnaire. Unlike in-person assessments, which require significant human capital, the entire project was developed and executed by a few individuals with a background in both medicine and computer programming. Using this system, we received a partial or full response from patients in 20 different states at an average of 355 (SD 335; range 5.61-1629) miles from our center. Furthermore, among patients who consented to the study, the questionnaire completion rate was very high, suggesting that collection of long-term parent-reported outcomes via electronic format is technically feasible and that the questionnaires chosen for the study were not overly burdensome to complete. Finally, among participants who completed the study, there was a strong willingness to repeat a future assessment and a high rate of participation in the second year.

Regarding limitations, it is important to remember that the results from this study represent screening examinations and cannot necessarily be used to diagnose atypical pediatric development. As the data are parent-reported, there is a possible bias toward either underreporting or overestimating a child’s capabilities [24]. Furthermore, when compared to nonresponders, the intertwin fetal growth discordance, a marker of placental insufficiency, and a risk factor for increased neonatal morbidity or mortality were lower in the patients who responded to the survey, which may bias the results toward a healthier population of individuals. Although most patients indicated they would consider providing pediatric records for evaluation, the study resources did not let us validate the questionnaires. It is, however, reassuring that the rates of atypical ASQ-3 screens in our cohort are comparable with rates of neurodevelopmental impairment, as assessed via Bayley Scales of Infant Development, which is administered in person, in several large European cohorts of patients treated for TTTS [8,26,27].

Patients in this study were identified retrospectively and were approached via email. It is possible that over time, patients’ contact information and email addresses changed, which may explain why nonresponders tended to have older children. A future study in which patients are enrolled prospectively and receive an in-person explanation of the study procedures and study purpose may lead to improved initial response rates.

Unfortunately, 32 patients were not eligible for the study due to not having an email on file. These patients, in addition to the nonresponders, may be less technically savvy and could represent a digital divide bias that favors participants who are more computer-literate or can afford a computer. For this study, we chose to use email as the method of survey distribution due to ease of use within REDCap. Potentially offering the survey via telephone, via post, or via alternative electronic media such as SMS text messaging may have improved response rates. In addition, the response rate may have been improved if participation had been incentivized, as participation was entirely voluntary.

The project was also only available in English. It is possible some patients for whom English was not their primary language received the study, although it is unlikely that they would have completed the questionnaires. Additional limitations include a lack of information regarding patient insurance status, which may be an important contributor to neurodevelopmental outcomes, and the absence of a control group for comparison. Finally, 11 patients who agreed to repeat the survey did not receive a repeat survey invitation in 2020 due to a technical coding error, which was not identified until after study completion. Furthermore, we were unable to reach several patients to confirm the results of atypical M-CHAT-R/F screens.

### Conclusions

This study represents the largest cohort of long-term outcomes reported in a population of patients treated with fetoscopic laser photocoagulation for TTTS in the United States. The novel use of computer programming and REDCap allowed us to automate the distribution, scoring, and generation of custom reports with ease and at relatively low cost. Future longitudinal studies in this population may benefit from prospective enrollment, incentivized participation, and survey distribution via alternative electronic methods such as SMS text messaging.

## Supplementary material

10.2196/60039 Multimedia Appendix 1 Fetal center questionnaire.

## Abbreviations

ASQ-3: Ages & Stages Questionnaires, Third Edition
CSQ: child status questionnaire
FC: fetal center
FCQ: fetal center questionnaire
FLP: fetoscopic laser photocoagulation
HIPAA: Health Insurance Portability and Accountability Act
M-CHAT-R/F: Modified Checklist for Autism in Toddlers, Revised With Follow-Up
MCDA: monochorionic diamniotic
REDCap: Research Electronic Data Capture
TTTS: twin-twin transfusion syndrome
TYQ: thank you questionnaire

## References

[1] Lewi L, Deprest J, Hecher K (2013). The vascular anastomoses in monochorionic twin pregnancies and their clinical consequences. Am J Obstet Gynecol.

[2] Sebire NJ, Snijders RJ, Hughes K, Sepulveda W, Nicolaides KH (1997). The hidden mortality of monochorionic twin pregnancies. Br J Obstet Gynaecol.

[3] Lewi L, Gucciardo L, Van Mieghem T (2010). Monochorionic diamniotic twin pregnancies: natural history and risk stratification. Fetal Diagn Ther.

[4] Berghella V, Kaufmann M (2001). Natural history of twin-twin transfusion syndrome. J Reprod Med.

[5] Senat MV, Deprest J, Boulvain M, Paupe A, Winer N, Ville Y (2004). Endoscopic laser surgery versus serial amnioreduction for severe twin-to-twin transfusion syndrome. N Engl J Med.

[6] Papanna R, Mann LK, Baschat AA (2015). Cervical length in prediction of preterm birth after laser surgery for twin-twin transfusion syndrome. Ultrasound Obstet Gynecol.

[7] Vanderbilt DL, Schrager SM, Llanes A, Hamilton A, Seri I, Chmait RH (2014). Predictors of 2-year cognitive performance after laser surgery for twin-twin transfusion syndrome. Am J Obstet Gynecol.

[8] van Klink JMM, Koopman HM, van Zwet EW (2014). Improvement in neurodevelopmental outcome in survivors of twin-twin transfusion syndrome treated with laser surgery. Am J Obstet Gynecol.

[9] Graeve P, Banek C, Stegmann-Woessner G, Maschke C, Hecher K, Bartmann P (2012). Neurodevelopmental outcome at 6 years of age after intrauterine laser therapy for twin-twin transfusion syndrome. Acta Paediatr.

[10] Gray PH, Poulsen L, Gilshenan K, Soong B, Cincotta RB, Gardener G (2011). Neurodevelopmental outcome and risk factors for disability for twin-twin transfusion syndrome treated with laser surgery. Am J Obstet Gynecol.

[11] Schou KV, Lando AV, Ekelund CK (2019). Long-term neurodevelopmental outcome of monochorionic twins after laser therapy or umbilical cord occlusion for twin-twin transfusion syndrome. Fetal Diagn Ther.

[12] Kowitt B, Tucker R, Watson-Smith D (2012). Long-term morbidity after fetal endoscopic surgery for severe twin-to-twin transfusion syndrome. J Pediatr Surg.

[13] Chmait HR, Chon AH, Korst LM, Putnam SP, Llanes A, Vanderbilt DL (2021). Child temperament outcomes after laser surgery for twin-twin transfusion syndrome. J Dev Behav Pediatr.

[14] Bergh EP, Donepudi R, Bell CS, Moise KJ, Johnson A, Papanna R (2020). Distance traveled to a fetal center and pregnancy outcomes in twin-twin transfusion syndrome. Fetal Diagn Ther.

[15] Gheorghe CP, Boring N, Mann L (2020). Neonatal outcomes and maternal characteristics in monochorionic diamniotic twin pregnancies: uncomplicated versus twin-to-twin transfusion syndrome survivors after fetoscopic laser surgery. Fetal Diagn Ther.

[16] Fox NS, Cohen N, Odom E (2018). Long-term outcomes of twins based on the intended mode of delivery. J Matern Fetal Neonatal Med.

[17] ASQ-3. Ages & Stages Questionnaires.

[18] Squires J, Bricker D, Potter L (1997). Revision of a parent-completed development screening tool: ages and stages questionnaires. J Pediatr Psychol.

[19] American Academy of Pediatrics Committee on Children With Disabilities (2001). Developmental surveillance and screening of infants and young children. Pediatrics.

[20] Dionne C, Squires J, Leclerc D (2004). Psychometric properties of a developmental screening test: using the Ages and Stages Questionnaires (ASQ) in Quebec and the US. J Intell Disabil Res.

[21] Skellern CY, Rogers Y, O’Callaghan MJ (2001). A parent-completed developmental questionnaire: follow up of ex-premature infants. J Paediatr Child Health.

[22] Kuban KCK, O’Shea TM, Allred EN, Tager-Flusberg H, Goldstein DJ, Leviton A (2009). Positive screening on the Modified Checklist for Autism in Toddlers (M-CHAT) in extremely low gestational age newborns. J Pediatr.

[23] Robins DL, Casagrande K, Barton M, Chen CMA, Dumont-Mathieu T, Fein D (2014). Validation of the Modified Checklist for Autism in Toddlers, Revised with Follow-up (M-CHAT-R/F). Pediatrics.

[24] (2020). Officer: manipulation of Microsoft Word and PowerPoint documents. R package version 0.3.13. R Project.

[25] Hovorka T (2018). WordR: rendering Word documents with R inline code. R package version 0.3.6. R Project.

[26] Lopriore E, Middeldorp JM, Sueters M, Oepkes D, Vandenbussche FPHA, Walther FJ (2007). Long-term neurodevelopmental outcome in twin-to-twin transfusion syndrome treated with fetoscopic laser surgery. Am J Obstet Gynecol.

[27] Lopriore E, Ortibus E, Acosta-Rojas R (2009). Risk factors for neurodevelopment impairment in twin–twin transfusion syndrome treated with fetoscopic laser surgery. Obstet Gynecol.

[28] NAFTNet centers. NAFTNet.

[29] Schneider EC, Shah A, Doty MM, Tikkanen R, Fields K, Williams II RD (2021). Mirror, mirror 2021—reflecting poorly: health care in the U.S. compared to other high-income countries. Commonwealth Fund.

[30] (2023). National uninsured rate reaches an all-time low in early 2023 after close of ACA open enrollment period (issue brief no. HP-2023-20). Office of the Assistant Secretary for Planning and Evaluation, U.S. Department of Health and Human Services.

[31] Furukawa MF, Machta RM, Barrett KA (2020). Landscape of health systems in the United States. Med Care Res Rev.

[32] Epic stork. Excite Health Partners.

[33] Meirte J, Hellemans N, Anthonissen M (2020). Benefits and disadvantages of electronic patient-reported outcome measures: systematic review. JMIR Perioper Med.

[34] Prasad S, Beg S, Badran D, Masciullo L, Huddy C, Khalil A (2024). Neurodevelopmental outcome in complicated twin pregnancy: prospective observational study. Ultrasound Obstet Gynecol.

[35] Tosello B, Blanc J, Haumonté JB, D’Ercole C, Gire C (2014). Short and medium-term outcomes of live-born twins after fetoscopic laser therapy for twin-twin transfusion syndrome. J Perinat Med.

[36] Korsakissok M, Groussolles M, Dicky O, Alberge C, Casper C, Azogui-Assouline C (2018). Mortality, morbidity and 2-years neurodevelopmental prognosis of twin to twin transfusion syndrome after fetoscopic laser therapy: a prospective, 58 patients cohort study. J Gynecol Obstet Hum Reprod.

[37] Salomon LJ, Ortqvist L, Aegerter P (2010). Long-term developmental follow-up of infants who participated in a randomized clinical trial of amniocentesis vs laser photocoagulation for the treatment of twin-to-twin transfusion syndrome. Am J Obstet Gynecol.

