# Supplementary material for: Use of Web-Based Surveys to Collect Long-Term Pediatric Outcomes in Patients With Twin-Twin Transfusion Syndrome Treated With Fetoscopic Laser Photocoagulation: Observational Study
Source: JMIR Pediatr Parent. 2024 Sep 11;7:e60039. doi: 10.2196/60039 (PMC11441452; doi:10.2196/60039)
Supplement: Multimedia Appendix 1 [file pediatrics-v7-e60039-s001.docx]

Appendix 1: Fetal Center Questionnaire

Fetal Center Questionnaire

1. Child’s date of delivery:
2. Child’s birthweight (lbs.ozs)
3. Child’s current weight (lbs.ozs)
4. Has your child been diagnosed with or ever been treated for:
   1. Colic (y/n)
   2. Asthma/Reactive airways (y/n)
   3. Any other chornic lung disease (y/n)
   4. Gastrointestinal reflux (y/n)
   5. Kidney (renal) disease (y/n)
   6. Heart (cardiac) disease (y/n)
   7. Necrotizing enterocolitis (y/n)
   8. Cerebral palsy (y/n)
   9. Difficulty with hearing (y/n)
   10. Diabetes (y/n)
   11. Speech/Language delay (y/n)
   12. Intellectual disability (y/n)
   13. Behavioral disorder (y/n)
5. Has your child ever required?
   1. Speech and/of Language Therapy (y/n)
   2. Occupational therapy (“OT”)
   3. Physical therapy (“PT”)
   4. Applied behavior analysis (“ABA”)
6. If your child is >= 2 years old
   1. AT OR AFTER the age of 2 years, has your peiatrician ever hand any concerns regarding your child’s:
      1. Height (too short): (y/n)
      2. Weight (too light): (y/n)
      3. Weight (too heavy): (y/n)
      4. Vision: (y/n)
      5. Hearing: (y/n)
      6. Motor skills: (y/n)
7. Please list any operations your child has undergone: (free text response)
8. Please list any medications your child takes regularly: (free text response)
9. In the last year, how many ER visits has your child required? (Dropdown: 0, 1, 2, 3, 4, 5 or more)
10. In the last year, how many hospital admissions has your child had?: (Dropdown: 0, 1, 2, 3, 4, 5 or more)
11. At what age (in months) did your child start crawling?: (Dropdown: 6 or less, 7-12, Greater than 12, Never crawled)
12. At what age (in months) did you child start walking independently?: (Dropdown: 9 or less, 10-24, > 24 months, Does not walk independently)
13. At what age (in months) did your child begin putting 2-3 word sentences together?: (free text)
14. If your child is school-age, what grade is he/she in (nursery, pre-k, kindergarten, 1^st^, 2^nd^, etc.): (Dropdown: My child does not attend school, Nursery school, Pre-K, Kindergarten, 1-8 grade)
15. Prior to entering school, did your child receive:
    1. Early Childhood Intervention (ECI) (birth to 3 year of age): (y/n)
    2. Head Start or other specialized pre-kindergarten programming (ages 3 until 5): (y/n)
    3. Individualized Education Plan (IEP)? (y/n)
    4. Section 504 plan: (y/n)
    5. Any specialized services or programming: (y/n)
    6. Admission, Review and Dismissal (ARD) or (IEP) meetings: (y/n)
